# Comparative iTRAQ proteomics revealed proteins associated with horn development in yak

**DOI:** 10.1186/s12953-018-0141-9

**Published:** 2018-07-24

**Authors:** Mingna Li, Xiaoyun Wu, Xian Guo, Pengjia Bao, Xuezhi Ding, Min Chu, Chunnian Liang, Ping Yan

**Affiliations:** Key Laboratory for Yak Breeding Engineering of Gansu Province, Lanzhou Institute of Husbandry and Pharmaceutical Sciences, Chinese Academy of Agricultural Sciences, Lanzhou, 730050 People’s Republic of China

**Keywords:** Horn bud, iTRAQ, Proteomics, Yak (*Bos grunniens*)

## Abstract

**Background:**

The practice of dehorning yak raises animal safety concerns, which have been addressed by selective breeding to obtain genetically hornless yak. The POLLED locus in yak has been studied extensively; however, little is known regarding the proteins that regulate horn bud development.

**Methods:**

A differential proteomic analysis was performed to compare the skin from the horn bud region of polled yak fetuses and the horn bud tissue of horned yak fetuses using isobaric tags for relative and absolute quantitation (iTRAQ) technology coupled with 2D LC-MS/MS.

**Results:**

One hundred differentially abundant proteins (DAPs) were identified. Of these, 29 were up-regulated and 71 were down-regulated in skin from the horn bud region of polled fetuses when compared to the horn bud tissue of horned fetuses. Bioinformatics analyses showed that the up-regulated DAPs were mainly associated with metabolic activities, while the down-regulated DAPs were significantly enriched in cell adhesion and cell movement activities.

**Conclusions:**

We concluded that some important proteins were associated with cell adhesion, cell motility, keratinocyte differentiation, cytoskeleton organization, osteoblast differentiation, and fatty acid metabolism during horn bud development. These results advance our understanding of the molecular mechanisms underlying horn development.

**Electronic supplementary material:**

The online version of this article (10.1186/s12953-018-0141-9) contains supplementary material, which is available to authorized users.

## Background

The yak is an important domesticated species found in the Qinghai-Tibetan Plateau and the adjacent alpine regions in China. Yak typically has a pair of persistent horns. However, hornless (polled) yaks exist in some populations. In modern husbandry systems, yak horns are a major cause of injury, and hornless yaks can be raised in higher densities [1]. Considering the animal safety concerns associated with dehorning [2], selective breeding of polled yak has become a promising alternative. Thus, studies on the genetic mechanism of horn differentiation and development have become increasingly popular.

Yak and cattle are related species belonging to the same genus. Despite anatomical and physiological differences, introgression at the genomic scale has occurred between them [3]. The polled phenotype is an autosomal dominant Mendelian trait that has been attributed to chromosome 1 on yak and cattle. In cattle, a 212-bp insertion-deletion causes hornlessness in most beef and dual-purpose breeds [4, 5], and an 80-kb duplication is associated with the polled phenotype in a majority of Holstein dairy cattle [4–6]. Both mutations are prevalent in some breeds (Limousin, Charolais, and Holstein), suggesting that introgression of the polled mutations is ongoing in those breeds [7]. In addition, a 219-bp duplication-insertion (*P*_219ID_) with a 7-bp deletion and a 6-bp insertion (*P*_1ID_) was recently identified as the mutation that determines hornlessness in Mongolian Turano cattle and yak [3]. These causal mutations do not include any known coding or regulatory region [3–5], thus adding to the complexity of identifying the genetic mechanism of the polled phenotype.

More recent efforts to identify the differential expression of genes and loci during horn bud development include quantitative RT-PCR, microarrays, and RNA-seq. Previously published data suggest that long noncoding RNA on BTA1 might be a prerequisite for horn bud formation. *FOXL2*, *RXFP2* and *OLIG2* might also be involved in horn bud differentiation [5, 7]. One of the most notable features when comparing transcriptional profiles of developing horn tissues against polled is the down regulation of genes encoding elements of the cadherin junction, which is a key structure involved in keratinocyte migration and subsequent horn development [8]. Currently, the genes primarily causative for bovine horn bud formation, and the underlying genetic mechanism of the polled trait remain unknown.

Horn development is the result of differentiation and remodeling of various tissues originating from two distinct germ layers: the ectoderm and the mesoderm [9]. Horn bud epidermis is thick and keratinized, whereas the underlying dermis and hypodermis are ossified. Histological changes have been described at different developmental stages in bovine fetuses. First, multiple layers of vacuolated keratinocytes are apparent in the first two to 6 months of gestation. At gestation day (gd) 212, the epidermis is well differentiated and keratinocytes are no longer vacuolated. Hair follicles below the horn bud are not present until gd 155. Thick nerve bundles develop in the dermis below the horn bud at gd 115 and become prominent shortly before birth [10]. Thus, exploring the molecular mechanism of horn developmental in yak in the early embryonic stages is an important first step.

Recent research has analyzed arsenic-induced skin keratosis proteomic profiles and identified several early molecular markers [11]. Previous findings have illustrated the usefulness of proteomic analysis of hair shaft corneocytes to test for genetic variation [12]. Hence, understanding the differential expression of proteins during horn bud development is of considerable importance to elucidate the recent genetic background of horn growth. The present work employed iTRAQ-based proteomics to characterize differences between the horn bud tissue of horned yak fetuses and the skin from the horn bud region of polled yak fetuses.

## Results

### Histological section of the horn bud in yak fetuses

**Fig. 1:**
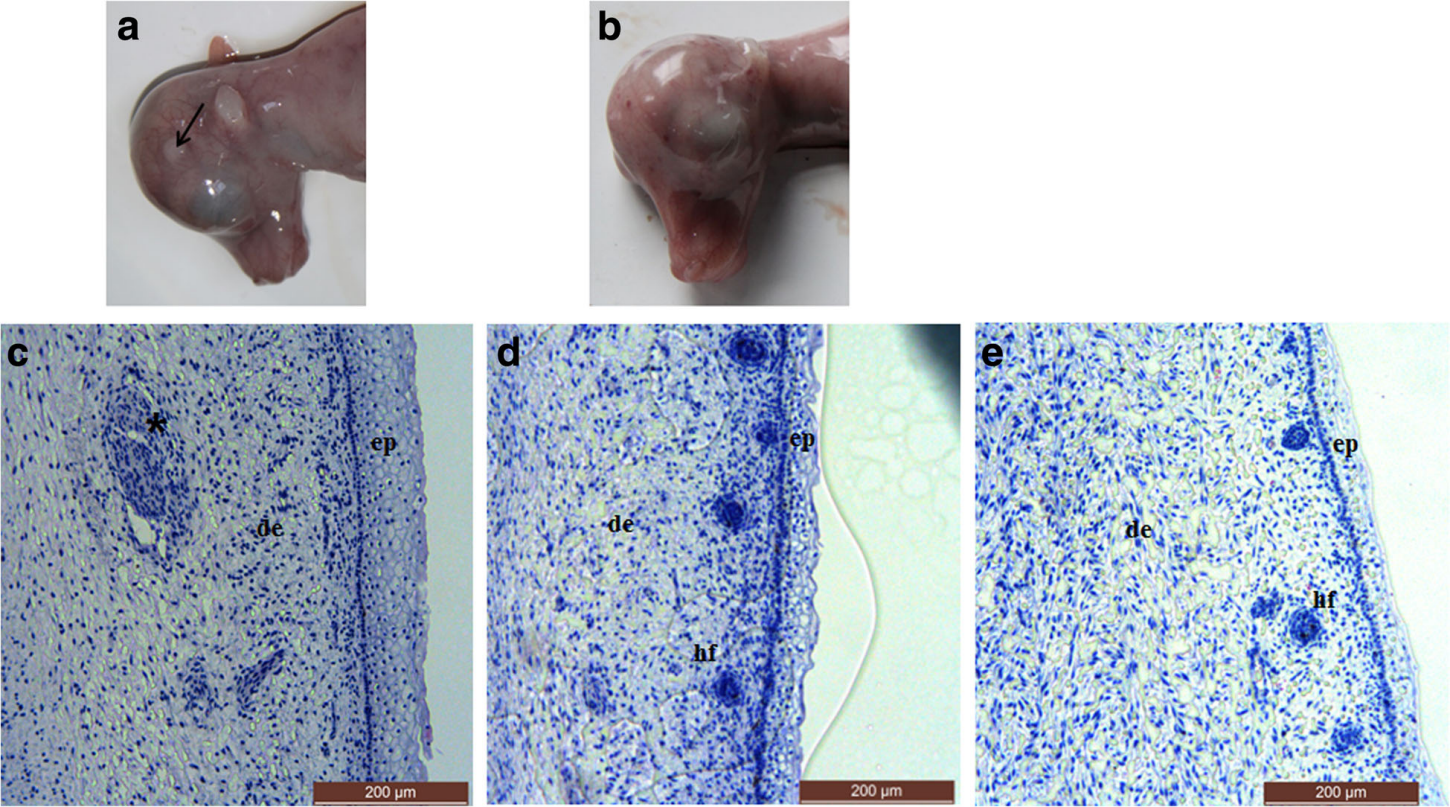
Feature of horn bud and frontal skin from horned and polled yak fetuses. **a** Macroscopic picture of a horn bud from a horned fetus. Black arrow indicates the area of indented skin. **b** Macroscopic picture of a polled fetus without indentation of the horn bud. **c** Histological section of a horn bud with multiple layers of vacuolated keratinocytes. Black star indicates the presence of clusters of dermal cells displaying different tissue. **d** Histological section of the “horn bud” region in polled yak fetus. **e** Histological section of frontal skin in horned yak fetus. Ep = epidermis, de = dermis, hf = hair follicles. The images in panels **c**, **d** and **e** were taken at 100 × magnification

Visual examination of horned fetuses (80–90 days post-coitum (dpc)) showed that the horn bud region was indented (Fig. 1a), while the comparable “horn bud” region in polled fetuses (80–90 dpc) consisted of normal skin and therefore could not be distinguished from the surrounding area (Fig. 1b). Histologically, the epidermis in the horn bud region showed supernumerary layers of vacuolated keratinocytes, while the epidermis of the frontal skin and of the horn bud region from polled fetuses was much thinner (Fig. 1c-e). Additionally, no hair follicles were present below the horn bud; while immature hair follicles in the hair germ stage were present in the superficial dermis of the frontal skin and polled fetal tissues. Finally, clusters of dermal cells were observed in the horn bud of the horned fetuses (Fig. 1c), such cells were not present in the dermis of frontal skin.

### Differentially abundant proteins (DAPs) identified by iTRAQ

To determine proteomic changes during yak horn bud development, we applied an iTRAQ-based quantitative proteomic approach. Global proteomic analysis in both skin from the horn bud region of polled fetuses and the horn bud tissue of horned fetuses (each group was composed of three biological replicates) detected a total of 5253 proteins (Additional file 1), each of which had at least one peptide identified with a minimum unused score that was ≥1.3, which indicates a > 95% confidence in correct sequence identification. The following analysis was based on proteins with a coefficient of variation value < 50%. Comparison of the horned and polled tissue proteome identified 100 proteins that met the analysis criteria (fold change of ≥1.5 or ≤ 0.67, *P* < 0.05), of which 29 proteins were up-regulated and 71 were down-regulated in skin from the horn bud region of polled fetuses when compared to the horn bud tissue of horned fetuses (Additional file 2). Among the down-regulated proteins, 23 proteins had fold-change values that were less than 0.5 (Additional file 2). The top four down-regulated proteins in polled yak compared to horned yak were keratin type II cytoskeletal 6B (KRT6B), keratin type I cytoskeletal 17 (KRT17), collagen alpha-1(XVIII) chain (COL18A1), and desmoplakin (DSP), which showed fold-change values of 0.0957, 0.2490, 0.2891 and 0.3010, respectively. In contrast, among the up-regulated proteins, 7 had fold-change values greater than 2.0 (Additional file 2). The serine/threonine-protein kinase (DCLK1) and polypeptide N-acetylgalactosaminyltransferase 16 (GALNTL1) were the top two up-regulated proteins with fold-change values of 3.2451 and 2.3611, respectively.

### PANTHER protein classification

**Fig. 2:**
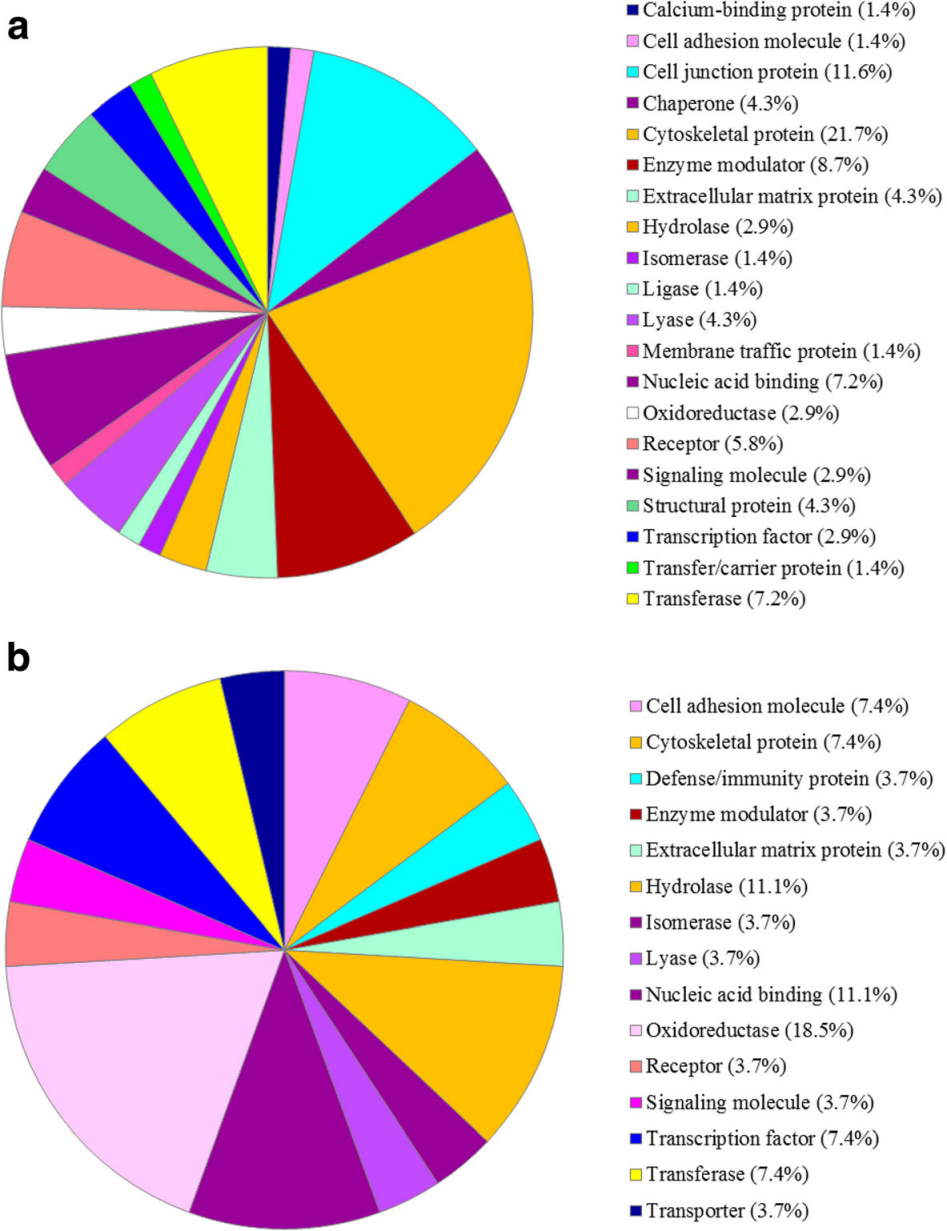
PANTHER classification of differentially expressed proteins in polled skin with respect to horn bud. **a** The classification of down-regulated proteins. **b** The classification of up-regulated proteins

The 71 DAPs which were down-regulated in skin from the horn bud region of polled yak fetuses when compared to the horn bud tissue of horned yak fetuses were categorized by the PANTHER (Protein Analysis Thorough Evolutionary Relationships) classification system into 20 protein classes, as illustrated in Fig. 2a. The majority of the proteins belonged to the cytoskeletal protein (21.7%), cell junction protein (11.6%), nucleic acid binding (7.2%) and transferase (7.2%) classes. In contrast, the 29 up-regulated DAPs were classified into 15 protein classes (Fig. 2b), in which most of the proteins belonged to oxidoreductase (18.5%), nucleic acid binding (11.1%), hydrolase (11.1%), transcription factor (7.4%), transferase (7.4%) and cytoskeletal protein (7.4%) classes. Thus, the PANTHER analysis showed that the majority of the up-regulated DAPs were related to metabolic activities, while that of down-regulated DAPs were related to cell junction, cytoskeletal formation, and other cell component organization.

### GO analysis of DAPs

**Fig. 3:**
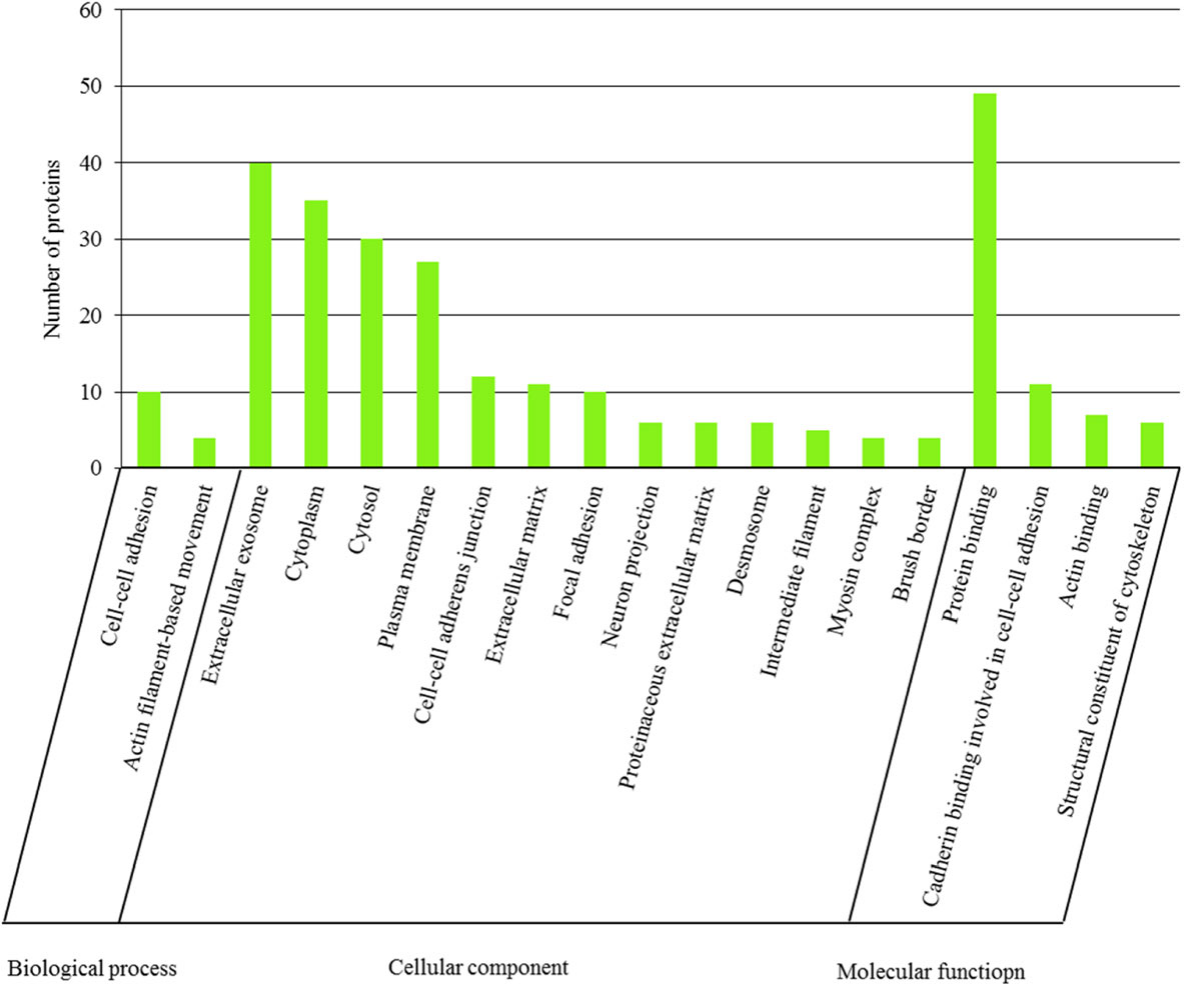
GO classification of down-regulated proteins in polled skin compared to the horn bud. The x-axis represents each GO terms. The y-axis represents the number of enriched proteins within each primary category

To identify enriched GO functional groups of DAPs, GO analyses were performed using DAVID. The Uniprot accession numbers of 97 DAPs were converted to human protein accession numbers due to the poor annotation of the bovine genome database. A total of 64 down-regulated DAPs between polled and horned skins were significantly enriched into 19 GO terms, including two biological process terms, 13 cellular component terms, and four molecular function terms (Fig. 3). Whereas no significantly enriched GO terms was found in up-regulated DAPs. Among the down-regulated DAPs, the enriched GO terms concerning biological process showed that DAPs were mainly associated with cell adhesion and cell movement activities. The top one listed enriched GO terms were “cell-cell adhesion”, followed by “actin filament-based movement”. Cellular component analyses showed that the targets were broadly distributed in different parts of the cell, with particular enrichment in the extracellular exosome, cytoplasm, cytosol and plasma membrane groups. In respect to the molecular function categories, the proteins were mainly enriched in the protein binding group, followed by the cadherin binding involved in cell-cell adhesion, actin binding, and structural constituent of cytoskeleton groups.

### Pathway enrichment analyses of DAPs

**Fig. 4:**
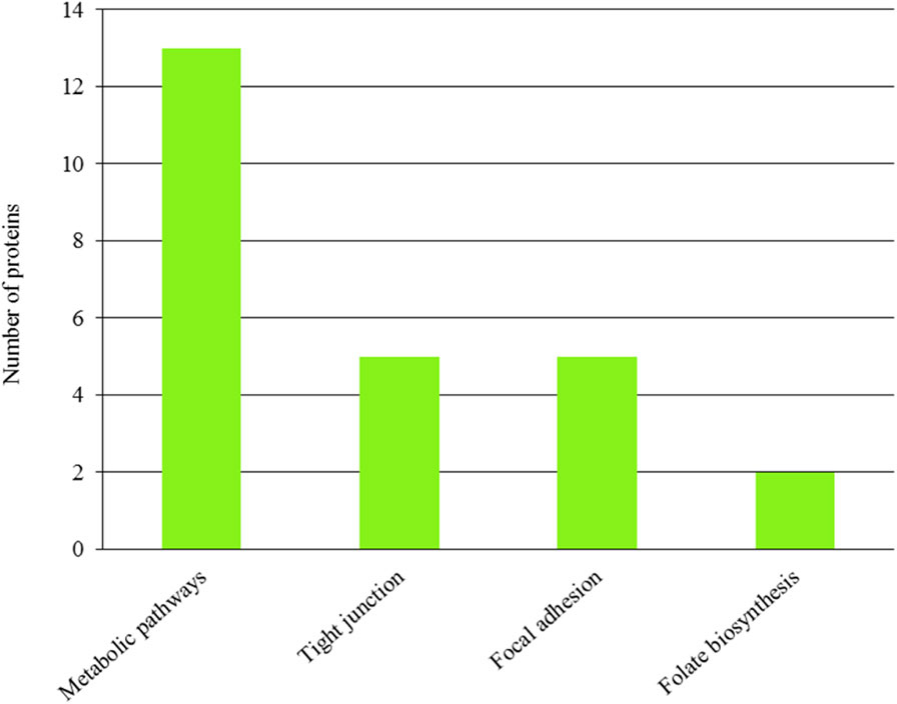
KEGG pathway analysis of differential expressed proteins in polled skin with respect to horn bud. The x-axis shows each of the enriched pathways. The y-axis represents the number of differentially abundant proteins (DAPs) in each primary category

DAPs were mapped to the reference pathway in the KEGG database to identify the biological pathways operating during horn bud development. Among the 100 identified DAPs, only 56 DAPs had a KEGG Orthology (KO) ID, and four pathways were significantly enriched (Fig. 4). The metabolic pathways, tight junction, focal adhesion, and folate biosynthesis pathways were significantly enriched. Thus, the four pathways were primarily associated with metabolic activities (metabolic pathway and folate biosynthesis) and cellular processes (tight junction and focal adhesion).

### Protein-protein interaction (PPI) analysis of DAPs

**Fig. 5:**
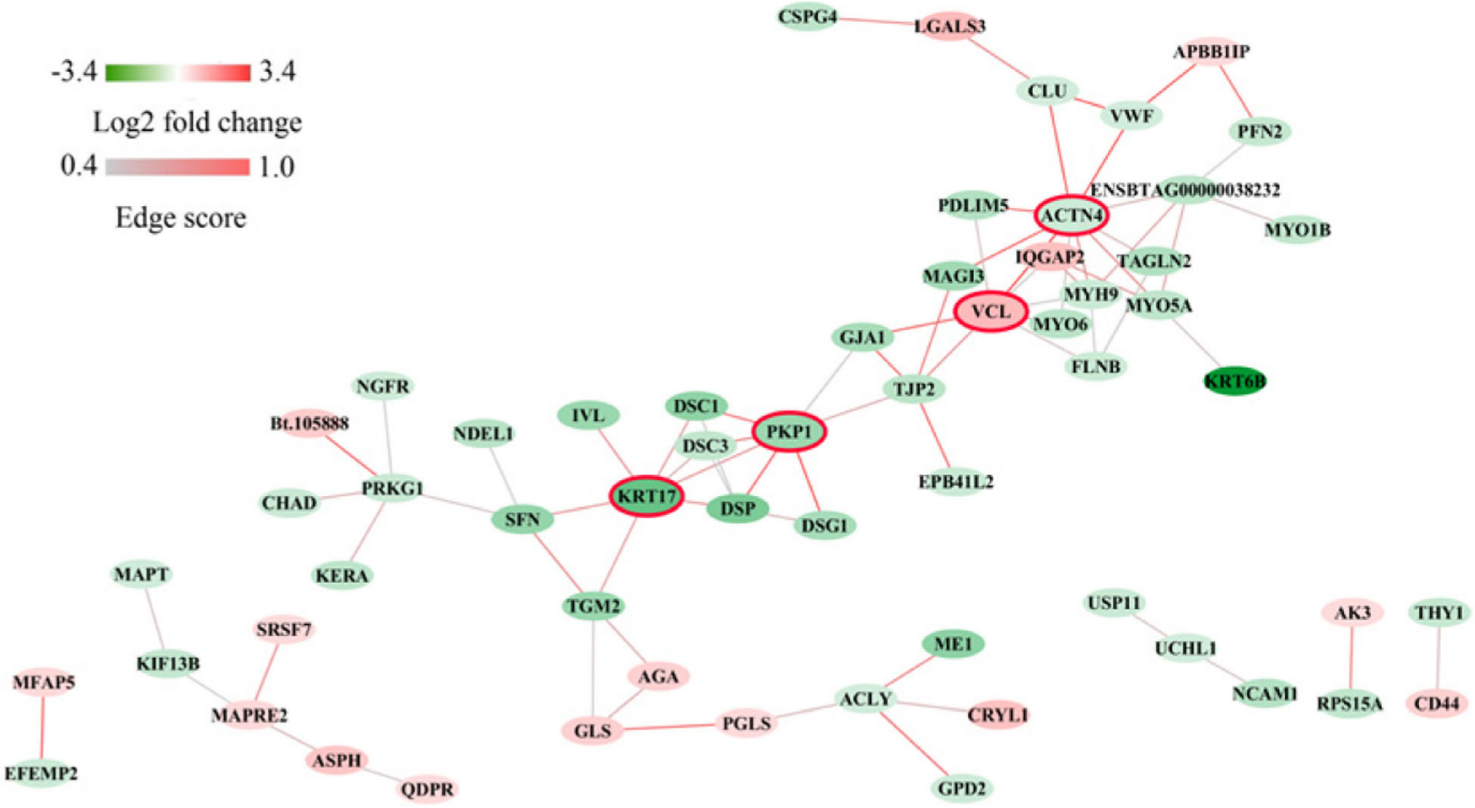
The protein-protein interaction network generated with STRING and differentially abundant proteins were visualized with Cytoscape. The red node indicates up-regulation and green node indicates down-regulation of DAPs. Proteins that are associated with each other are linked by an edge. The color of the edge indicates the combined interaction score (edge score). The nodes with red borders represent hub proteins

The PPI network of the DAPs identified in our study was analyzed using the Search Tool for the Retrieval of Interacting Genes/Proteins 10 (STRING 10) database. By removing unconnected proteins and self-loops, the resulting PPI network contained 59 protein nodes and 75 edges (Fig. 5). Proteins with higher connectivity than others in the network were referred to as “hubs”. These hubs may play crucial roles in the regulation of the network. PPI analysis revealed the top four hub proteins: alpha-actinin 4 (ACTN4), vinculin (VCL), keratin type I cytoskeletal 17 (KRT17), and plakophilin 1 (PKP1).

### Western blot analyses

**Fig. 6:**
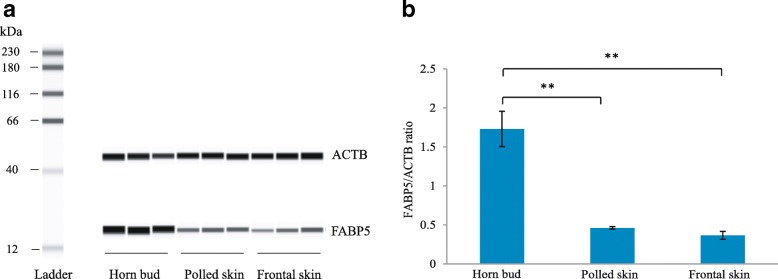
Validation of iTRAQ data by Western blot analyses. **a** The Western blot bands of FABP5. **b** The relative levels of FABP5 (normalized to ACTB). Error bars indicate standard deviations. The asterisks show significant differences (*P* < 0.01)

Western blot analyses were performed to verify the results obtained from the iTRAQ proteomics experiment. The expression level of fatty acid-binding protein 5 (FABP5) on horn bud tissue of horned fetuses was significantly higher than levels found in the skin from horn bud region of polled fetuses and the frontal skin of horned fetuses (*P* < 0.01) (Fig. 6a-b). The ratio of FABP5 expression level in polled yak compared to those in horned yak from the western blot was about 0.2665 (0.4612/1.7309) (Additional file 3), which is lower than that of ratio 0.3950 from iTRAQ (Additional file 2). However, the results showed the same trend in western blot and iTRAQ. These data support the results obtained from iTRAQ.

## Discussion

In the histological analyses, we compared the horn bud region skin of polled fetuses, horn bud and frontal skin of horned fetuses from 80 to 90 dpc yak. Histological sections of the horn buds displayed three main morphological changes: multiple layers of vacuolated keratinocytes, absence of immature hair follicle, and clusters of dermal cells displaying different tissue. These findings are consistent with observations in previous studies in cattle [10], in which clusters of dermal cells were demonstrated as thick nerve bundles [10], it is speculated that such cells in Fig. 1c might be differentiating nerve bundles. However, no evidence of osteoblast and chondroblast differentiation was found in this study. These changes may occur later in fetal life or after birth. Determining the mechanisms that regulate fetal horn bud development and how they are differently employed in polled yak is of vital importance for understanding the biology of horn ontogenesis.

Some DAPs may be associated with cell adhesion. Desmosomes provide cells with binding domains for intermediate filaments of the cytokeratin network [13]. Desmosomes from neighboring cells are connected to one another by specialized cadherin adhesion molecules called desmogleins (DSGs) and desmocollins (DSCs) [14]. Plakophilins (PKPs), one of the armadillo family members, bind to desmosomal cadherins and indirectly link the desmosomal cadherins to intermediate filaments. In turn, this binding recruits the cytolinker DSP, which binds keratin intermediate filaments to the desmosomal plaque [14, 15]. Desmoglein 1 (DSG1) is first expressed in the suprabasal epithelial layers and becomes more concentrated in the upper and most differentiated layer where it promotes cell adhesion and keratinocyte differentiation [15, 16]. Targeted disruption of the desmocollin 1 (*DSC1*) gene results in characteristic epidermal defects in transgenic mice, illustrating the importance of this molecule in maintaining normal intercellular adhesion [17]. Knockout of *PKP1* results in the loss of keratinocyte adhesion and subsequent abnormal epidermal differentiation [18]. Studies have demonstrated that DSP is essential for the maturation of adherens junctions [19]. Consistent with this, the results described here showed increased expression of DSG1, DSC1, desmocollins 3 (DSC3), PKP1, and DSP in the horn bud, suggesting that strong adhesion is required in multiple layers of keratinocyte differentiation. Therefore, these proteins are important for the skin differentiation process.

Some DAPs may be associated with cytoskeleton organization and keratinocyte differentiation. Keratins are structurally related proteins that form intermediate filaments and occur primarily in epithelial cells. Together with actin microfilaments and microtubules, intermediate filaments form the cytoskeleton of epithelial cells. In addition to providing mechanical support, keratins fulfill a variety of important functions, including maintenance of cellular integrity, regulation of cell proliferation and migration, and protection from apoptosis [20]. Mutation in keratin can cause a broad range of epithelial diseases [21, 22]. Mutations in *KRT6B* and *KRT17* are responsible for pachyonychia congenita [21]. A genome-wide gene expression profile of adalimumab-treated psoriatic skin found that *KRT6B* was one of the top differentially expressed genes [23]. Keratin 6C is a biomarker for early diagnosis in arsenic-induced skin keratosis [11]. KRT17 is expressed under various pathological conditions, including psoriasis and cutaneous allergic reactions but is not found in healthy epidermis [24]. It might be speculated that KRT6B and KRT17 play an important role in cornification of skin. In this study, the results showed that KRT6B and KRT17 are highly expressed in the horn bud, which is consistent with the structure of multiple layers of keratinocytes in the horn bud. Since abnormal expression of KRT6B and KRT17 may affect their structural role in the epidermis, these proteins might represent a biomarker for skin disorders. Moreover, KRT6B and KRT17 may contribute to horn bud development.

As keratinocytes migrate towards their ultimate destination in the upper epidermis, they undergo a unique process of cornification (or keratinization), which is distinct from apoptosis [13, 25]. A series of keratinocyte proteins, including involucrin (IVL) and small proline rich proteins (SPRs), are synthesized and subsequently crosslinked by transglutaminases to reinforce the cornified envelope [25]. Our results showed higher expression of IVL and small proline rich protein 9 (SPRR9) in horn buds, which is consistent with the constituent of more keratinocytes in horn bud. All of these proteins are related to epidermal and keratinocyte differentiation and may thus be important constituents of the horn bud.

Some DAPs may be associated with cell motility. In addition to cytokeratins and their contributions to the intermediate filament network, the other important structural components of living cells are actin filaments, which contribute to cell motility, polarity, and adhesion. Cell migration depends on the conversion of chemical energy into mechanical propulsion, which is produced by actin polymerization [26]. This activity primarily involves dynamic interactions between the extracellular matrix and the actin cytoskeleton mediated through integrins. Several integrin-associated proteins are known to connect the integrins with the actin cytoskeleton, and one such example is alpha-actinin [8, 27]. The expression of ACTN4 is down-regulated in polled versus horned animals, and ACTN4 is vital for cell migration [28, 29]. Inhibition of ACTN4 function appears to attenuate cancer metastasis [30]. In addition, an important component of the actin cytoskeleton is myosin. Myosin binds to filamentous actin and produces physical forces by hydrolyzing ATP to move along actin tracks [31]. Several isoforms of the class II non-muscle myosin family (myosin-9; MYH9), unconventional myosin (myosin-Ib; MYO1B), myosin-VI (MYO6), and myosin-Va (MYO5A) showed higher expression in horned compared to polled tissues. We speculate that ACTN4 and myosin family members may be associated with vesicle cytoskeletal movement.

Some DAPs may be associated with osteoblast differentiation. The bony core originates from a separated center of ossification located in the dermis and hypodermis of the horn bud [9]. In our study, no signs of osteoblasts or chondroblasts were present in any of the fetal tissue samples; however, some differentially abundant proteins were identified to be involved in osteoblast differentiation. Fibrillin 2 (FBN2) regulates tissue elasticity. Mutations in the FBN2 gene are associated with congenital contractual arachnodactyly (CCA), a condition characterized by dolichostenomelia, pectus deformities, kyphoscoliosis, and congenital contractures [32]. Gap junction alpha 1 protein (GJA1) is one of the most potent gap junction proteins important for osteoblast differentiation and bone formation. Previous studies have confirmed that mutations in *GIA1* are responsible for oculodentodigital dysplasia (ODDD), characterized by developmental abnormalities of the face, eyes, limbs, and dentition [33, 34]. The differential expression of the above proteins may contribute to the ossification of the dermis and hypodermis in the later stage of yak horn bud development.

Some DAPs may be associated with fatty acid metabolism. Fatty acid-binding proteins are hypothesized to serve as lipid shuttles that solubilize hydrophobic fatty acids and deliver them to appropriate intracellular sites [35]. The epidermal FABP (E-FABP), also known as FABP5, was first identified in human epidermis. FABP5 is predominantly expressed in keratinocytes and actively proliferating tissue, such as that of psoriasis [36]. Although the mechanisms underlying the up-regulation of FABP5 in keratinocytes and psoriasis are not completely understood, previous studies suggest that FABP5 may have important roles in the regulation of fatty acid metabolism during keratinocyte differentiation in the pathogenesis of psoriasis [35]. We found FABP5 was significantly increased in horn bud epidermis compared to that in normal skin, consistent with previous reports describing arsenic-induced skin keratosis [11]. It has been speculated that FABP5 expression is increased in response to increased lipid trafficking [35], which may be associated with keratinocyte proliferation and epidermis differentiation in horn bud.

## Conclusions

In conclusion, to the best of our knowledge, this is the first study to provide proteomic data to assess prenatal horn bud development in yak. The results showed that several key proteins involved in different cell processes, including cell adhesion, cell motility, keratinocyte differentiation, cytoskeleton organization, osteoblast differentiation and fatty acid metabolism. These processes have indispensable roles during horn development in yak. These results contribute to the understanding of the genetic and molecular mechanisms underlying horn development.

## Methods

### Sample collection and preservation

All experiments were carried out in strict accordance with the recommendations in the Guide for the Care and Use of Laboratory Animals of the National Institutes of Health. Polled (*n* = 3) and horned (n = 3) fetuses of Datong yak were obtained from a slaughterhouse located at the Datong Yak Farm of Qinghai Province of China. The fetuses were estimated to be 80–90 dpc, based on the crown-rump length of fetus [37]. Samples of the horn buds and the frontal skin were collected using scalpel (Fig. 1a). The same region was collected in polled fetuses (Fig. 1b). The left horn buds and forehead skin were fixed with 4% paraformaldehyde for histological analyses, and the right horn buds were frozen in liquid nitrogen until further use.

### Histological preparation

The hematoxylin and eosin staining method was used to examine the histological characteristics of horn buds and forehead skin from fetal yaks. Tissue fragments were dehydrated in a series of graded ethanol solutions, cleared with xylene, and embedded in paraffin. Microtome sections (5 μm, Leica RM2255) were stained with hematoxylin and eosin (Solarbio, Beijing, China). Stained sections were observed using a Leica DM5500.

### Protein preparation

The horn bud samples from three horned and polled fetuses were frozen in liquid nitrogen, homogenized to powders, and proteins were extracted with radioimmunoprecipitation assay (RIPA) lysis buffer (50 mM Tris pH 7.4, 150 mM NaCl, 1% Triton X-100, 0.1% SDS, 1% sodium deoxycholate) containing 1 mM PMSF. Homogenates were sonicated 10 times at 20 W with 1 min per pulse. Samples were incubated on ice for 20 min and supernatants were sonicated an additional 6 times at 20 W with 1 min per pulse. Samples were then centrifuged at 12,000 rpm for 20 min at 4 °C. The supernatant was transferred to a new tube and quantified using the Bradford method.

Approximately 200 μg of proteins were reduced with 5 mM TCEP at 60 °C for 1 h. Subsequently, 10 mM methyl methanethiosulfonate (MMTS) was added to block cysteine residues; homogenates were incubated for 30 min in the dark and then filtered using 10 kDa MWCO Nanosep® centrifugal filters at 12,000 rpm for 20 min (Pall corporation, New York, USA). The proteins were dissolved in 100 μL 0.25 M triethylammonium bicarbonate (TEAB; Sigma-Aldrich, St. Louis, MO, USA) and then washed and centrifuged (12,000 rpm for 20 min) three times. Proteins were adjusted to a final volume of 50 μL with 0.5 M TEAB prior to digestion with trypsin (Promega, Madison, WI, USA) overnight at 37 °C at a 1:50 trypsin-to-protein mass-ratio.

### iTRAQ labeling and high pH reverse phase separation

After digestion with trypsin, peptides were dried by vacuum centrifugation and then reconstituted in 50 μL 0.5 M TEAB. Fifty μL samples (100 μg total protein) were transferred to new tubes and labeled according to the manufacturer’s protocol with 8-plex iTRAQ reagent (Applied Biosystems, CA, USA). Briefly, one unit of iTRAQ reagent was thawed and reconstituted in 150 μL isopropanol. The peptide samples from three horned yak fetuses were labeled with iTRAQ tags of 113, 115 and 118, and those from three polled yak fetuses were labeled with iTRAQ tags of 114, 117 and 119. After incubation at room temperature for 2 h, the labeled peptide mixtures were pooled and dried by vacuum centrifugation.

The first dimensional reverse phase separation was performed using a Dionex Ultimate 3000 Rapid Separation LC Systems (Dionex, Sunnyvale, California, USA). The iTRAQ-labeled peptide mixtures were reconstituted with 200 μL buffer A (20 mM HCOONH_4_, 2 M NaOH, pH 10) and loaded onto a 150 × 2.00 mm Gemini-NX C18 column (Phenomenex, CA, USA). The columns were equilibrated with 95% buffer A and 5% buffer B (20 mM HCOONH^4^, 2 M NaOH, 80% acetonitrile (ACN), pH 10) for 30 min. The peptides were eluted at a flow rate of 200 μL/min with a gradient of 5% buffer B for 10 min, 5–15% buffer B for 5 min, 15% buffer B for 10 min, 15–50% buffer B for 45 min, 50% buffer B for 10 min, and 50–100% buffer B for 10 min. The system was maintained at 100% buffer B for 10 min. Elution was monitored by measuring the absorbance at 214 nm, and fractions were collected every 1 min. The fractions were pooled as required.

### LC-MS/MS analyses using the Orbitrap Q Exactive

The fractions from the first dimension were resuspended in 0.1% FA and 2% ACN. Fractions were centrifuged at 12,000 rpm for 20 min at 4 °C, and the supernatants were loaded on a Thermo Dionex Ultimate 3000 RSLC Nano system (Dionex, Sunnyvale, California, USA) by the autosampler onto a C18 reverse phase column (100 μm i.d., 10 cm long, 3 μm resin; Michrom Bioresources, Auburn, CA). Peptides were eluted with a linear gradient (5 to 40%) of buffer B (0.1% FA, 95% ACN) for 70 min at 300 nL/min.

The peptides were subjected to nanoelectrospray ionization followed by tandem mass spectrometry (MS/MS) using an Orbitrap Q EXACTIVE spectrometer (Thermo Fisher Scientific, CA, USA) coupled online to the HPLC. Intact peptides were detected in the Orbitrap at a mass resolution of 70,000. Peptides were selected for MS/MS and further fragmented by high-energy collision-induced dissociation using a normalized collision energy setting of 30 eV; ion fragments were detected in the Orbitrap at a mass resolution of 17,500. A data-dependent procedure that alternated between one MS scan and a 15 MS/MS scan with a dynamic exclusion duration of 15 s was then performed. Automatic gain control was used to optimize the spectra generated by the Orbitrap. The targeted automatic gain control value for MS1 was 3e6 and 1e5 for MS2. The m/z scan range was 350–2000 Da.

### Data analysis

Protein identification and quantification were performed with ProteinPilot™ Software 5.0 (Applied Biosystems) using the Paragon algorithm (5.0.1.0, 4874) for peptide identification. Specific search parameters were applied: (1) detected protein threshold: 0.05; (2) competitor error margin: 2.00; (3) revision number: 4895; (4) instrument: Orbi MS (1–3 ppm), Orbi MS/MS; (5) sample type: iTRAQ 8plex (Peptide Labeled); (6) cysteine alkylation: MMTS; (7) digestion: trypsin; (8) special factors: none; (9) ID focus: biological modifications; (10) search effort: thorough ID; (11) false discovery rate (FDR) analysis: Yes; (12) user modified parameter files: no. The required MS/MS spectra were searched against BOVIN_2016.2.18_uniprot.fasta (http://www.uniprot.org/proteomes/UP000009136) database. Unique proteins with at least one unique peptide were identified and the FDR was set to < 0.01 for the identification of both peptides and proteins. A peptide confidence level of 95% or an unused confidence score > 1.3 was used as the qualification criteria. Relative quantification of proteins was calculated based on the ratio of peak areas from MS/MS spectra. Differentially abundant proteins were determined using Student’s t-test. Proteins with a < 0.05 *P*-value and a fold change ≥1.5 were considered up-regulated and a fold change ≤0.67 was considered down-regulated.

### Bioinformatic analysis

The PANTHER classification system (http://www.pantherdb.org/index.jsp) was employed to identify protein classes [38]. The DAPs identified in this study were converted to human orthologous proteins, and enrichment analyses were also performed to identify GO terms that were significantly enriched using the DAVID software (http://david.abcc.ncifcrf.gov/) [39, 40]. The Benjamin-Hochberg adjustment was used for multiple test correction of enriched *P* values. To enrich for the statistically significant biological pathways of the identified proteins, the KEGG orthology-based annotation system (KOBAS, http://kobas.cbi.pku.edu.cn) was used [41]. The pathway enrichment was conducted by a hypergeometric statistics test. The Benjamin-Hochberg FDR correction was used to correct the probability values, and only those corrected values at *P* < 0.05 were considered significantly enriched pathways. PPI networks were built using the publicly available program, STRING [42]. Each interaction had a combined score (edge score), which represented the reliability of the interaction between proteins. The Cytoscape tool was utilized to visualize the interaction networks [43].

### Western blot analysis

Western blot analyses of skin tissues were performed using a Simple Western™ system (ProteinSimple, USA). Another polled (*n* = 3) and horned (n = 3) fetuses (80–90 dpc) of Datong yak were obtained from the same place. The horn bud and the frontal skin were collected at the same developmental stage as the fetal yak. The corresponding regions of the horn buds were collected in polled fetuses. Total proteins for all samples were extracted, mixed with Simple Western Sample Buffer at a final concentration of 0.2 μg/mL, reduced and denatured. Anti-fatty acid binding protein 5 (ab84028; Abcam) diluted to 1:200 was used as the primary antibody. ACTB (ab8224, Abcam) was used as the loading control. The biotinylated MW ladder, prepared samples, primary and secondary antibodies (goat anti-rabbit secondary antibody, 042–206; goat anti-mouse secondary antibody, 042–205, ProteinSimple) and chemiluminescent substrate were dispensed in microliter volumes into the designated wells of 8 × 25 capillary cartridges. The Simple Western assay buffers, nanovolume capillaries, and the prepared assay plates were loaded into Wes™, which performs all assay steps automatically. The western blot experiments were performed with three biological replicates. Statistical analyses were performed by one-way ANOVA using SPSS18.0.

## Additional files

Additional file 1: Total proteins identified in the iTRAQ-based experiment. (XLS 657 kb)

Additional file 2: Total number of differentially abundant proteins determined by iTRAQ. (XLS 73 kb)

Additional file 3: The data on Western blot analysis. (XLS 9 kb)

## Abbreviations

ACTN4: Alpha-actinin 4
CCA: Congenital contractual arachnodactyly
COL18A1: Collagen alpha-1(XVIII) chain
DAPs: Differentially abundant proteins
DCLK1: Serine/threonine-protein kinase
dpc: Days post-coitum
DSC1: Desmocollin 1
DSC3: Desmocollins 3
DSCs: Desmocollins
DSG1: Desmoglein 1
DSGs: Desmogleins
DSP: Desmoplakin
E-FABP: Epidermal FABP
FABP5: Fatty acid-binding protein 5
FBN2: Fibrillin 2
FDR: False discovery rate
GALNTL1: Polypeptide N-acetylgalactosaminyltransferase 16
gd: Gestation day
GJA1: Gap junction alpha 1 protein
iTRAQ: Isobaric tags for relative and absolute quantitation
IVL: Involucrin
KO: KEGG Orthology
KOBAS: KEGG orthology-based annotation system
KRT17: Keratin type I cytoskeletal 17
KRT6B: Keratin type II cytoskeletal 6B
MMTS: Methyl methanethiosulfonate
MYH9: Myosin-9
MYO1B: Myosin-Ib
MYO5A: Myosin-Va
MYO6: Myosin-VI
ODDD: Oculodentodigital dysplasia
PANTHER: Protein Analysis Thorough Evolutionary Relationships
PKP1: Plakophilin 1
PKPs: Plakophilins
PPI: Protein-protein interaction
RIPA: Radioimmunoprecipitation assay
SPRR9: Small proline rich protein 9
SPRs: Small proline rich proteins
STRING: Search Tool for the Retrieval of Interacting Genes/Proteins
TEAB: Triethylammonium bicarbonate
VCL: Vinculin
